# Efficacy and safety of nanoparticle-albumin-bound paclitaxel compared with solvent-based taxanes for metastatic breast cancer: A meta-analysis

**DOI:** 10.1038/s41598-019-57380-0

**Published:** 2020-01-17

**Authors:** Hwaryeon Lee, Sohyun Park, Ji Eun Kang, Hee Min Lee, Sun Ah Kim, Sandy Jeong Rhie

**Affiliations:** 1 Graduate School of Converging Clinical & Public Health, Ewha Womans University, Seoul, 03760 Republic of Korea; 2 Department of Pharmacy, Ewha Womans University Mokdong Hospital, Seoul, 07985 Republic of Korea; 3 Graduate School of Pharmaceutical Sciences, Ewha Womans University, Seoul, 03760 Republic of Korea; 4 College of Pharmacy, Ewha Womans University, Seoul, 03760 Republic of Korea; 5 Department of Pharmacy, National Medical Center, Seoul, 04564 Republic of Korea; 6 Division of Life & Pharmaceutical Science, Ewha Womans University, Seoul, 03760 Republic of Korea; 7 Department of Pharmacy, Ewha Womans University Seoul Hospital, Seoul, 07985 Republic of Korea

**Keywords:** Breast cancer, Cancer therapy

## Abstract

The curative effects of nanoparticle albumin-bound (nab)-paclitaxel in the first-line treatment of metastatic breast cancer (MBC) are still controversial, with even more after the removal of marketing approval of indication of bevacizumab. Five electronic databases and the related resources were searched for eligible randomized clinical trials (RCTs) without year and language restrictions to perform a meta-analysis. The studies were comparing the efficacy and safety between nab-paclitaxel chemotherapy versus solvent-based (sb)-taxanes chemotherapy such as sb-paclitaxel and docetaxel. The primary end points were overall response rate (ORR) and disease control rate (DCR). Secondary end points were progression-free survival (PFS), overall survival (OS), adverse events (AEs), and dose discontinuation rate (DDR). Five RCTs (1,554 patients) were finally identified from 1,902 studies. When compared to sb-paclitaxel, nab-paclitaxel showed significant beneficial effects in terms of ORR (OR 2.39, 95% CI 1.69–3.37, p < 0.001), DCR (OR 1.89, 95% CI 1.07–3.35, p = 0.03), and PFS (HR 0.75, 95% CI 0.62–0.90, p = 0.002). Nab-paclitaxel also showed significantly longer OS (HR 0.73, 95% CI 0.54–0.99, p = 0.04) than docetaxel. AEs and DDR were comparable between the two arms. Using nab-paclitaxel could significantly improve efficacy with comparable toxicities in the treatment of MBC.

## Introduction

Nanoparticle albumin-bound (nab) paclitaxel is solvent free and employs a novel delivery mechanism for paclitaxel to tumors^1^. Although nab-paclitaxel was initially developed to minimize the toxic effects of taxane treatment, several early clinical trials demonstrated that nab-paclitaxel was also more effective than the conventional solvent-based (sb) paclitaxel in the treatment of metastatic breast cancer^2–5^.

However, recent randomized controlled trials (RCTs) have suggested that nab-paclitaxel chemotherapy is not as efficacious as sb-taxanes, such as sb-paclitaxel and docetaxel, and that nab-paclitaxel is often associated with more frequent adverse events^6^. For example, Rugo *et al*. showed that nab-paclitaxel was not superior to sb-paclitaxel (progression-free survival [PFS] 9.3 months vs 11 months, hazard ratio [HR] 1.20, 95% confidence interval [CI] 1.00–1.45, p = 0.054). Results were concordant with overall survival (OS), and time to treatment failure was significantly shorter in the nab-paclitaxel arm vs the sb-paclitaxel arm^6^. Hematologic and non-hematologic toxicity, including peripheral neuropathy, was more prevalent in the nab-paclitaxel arm, which also had more frequent and earlier dose reductions^6^.

After these conflicting results were generated, Liu *et al*. conducted a first meta-analysis of randomized clinical trials to evaluate the efficacy and toxicity of nab-paclitaxel compared with sb-paclitaxel and docetaxel in the treatment of metastatic breast cancer (MBC), and they concluded that nab-paclitaxel was associated with more frequent sensory neuropathy, but only equivalent survival and possibly higher overall response for only some specific subgroups^7^. They also cited that nab-paclitaxel chemotherapy was more expensive than conventional sb-taxane chemotherapy^7^. The findings of Liu *et al*. seemed to be affected largely by the phase III trial of Rugo *et al*. In this trial, they administered bevacizumab (10 mg/kg, day1, day15), along with nab-paclitaxel (for the experimental arm) or sb-paclitaxel (for the control arm) to chemotherapy-naive MBC patients.

However, the US Food and Drug Administration (FDA) withdrew its approval of bevacizumab in combination with paclitaxel in the treatment of MBC in December 2010^8^. Therefore, it might be reasonable to assume that the combined bevacizumab regimen may obscure the taxane effect, meaning that the previous meta-analysis may not reveal the real efficacy and safety of nab-paclitaxel and sb-taxanes. To prevent information loss due to summarization, it is important to include all relevant and eligible randomized trials in a meta-analysis^9^. However, we believe that it is also reasonable to exclude data that are inappropriate.

In this updated meta-analysis, therefore, we conducted a subgroup analysis aimed to concentrate on the comparison of medications without bevacizumab which compared between nab-paclitaxel and sb-taxanes. With this approach, we can concentrate solely on the efficacy and safety of nab-paclitaxel compared with conventional sb-taxanes. We analyzed two more efficacy variables, disease control rate (DCR) and PFS, with an additional recent randomized trial^10^.

## Results

### Search results

**Figure 1:**
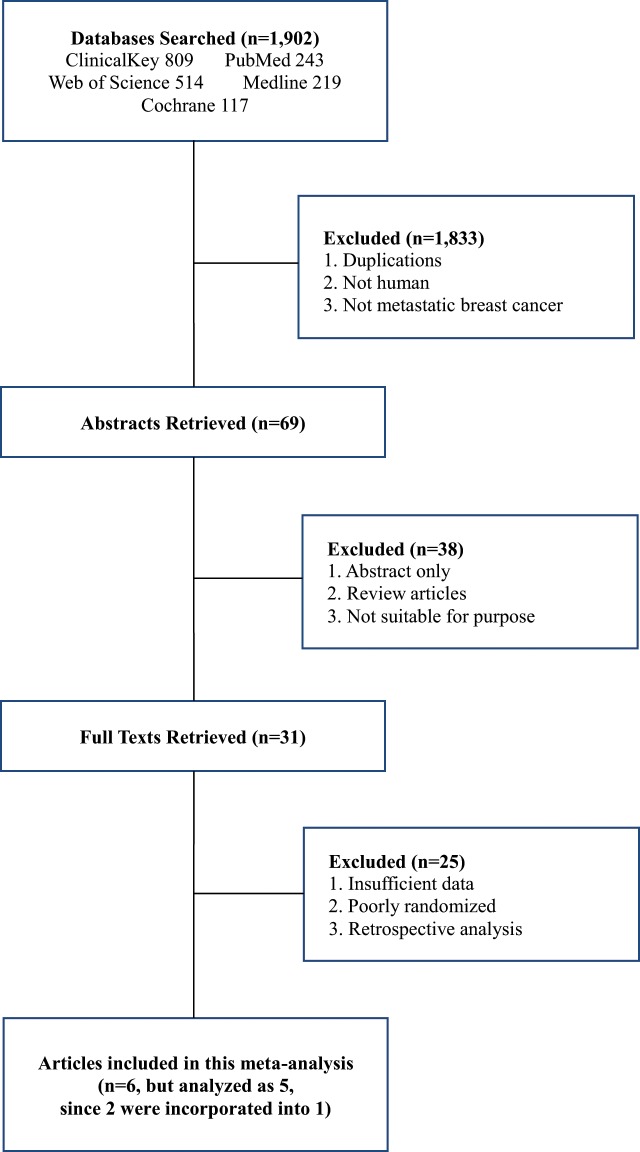
PRISMA Flow diagram of the study selection.

After screening 1,902 studies chosen from electronic databases and manual searches, we finally included six eligible randomized trials^2–6,10^ for this meta-analysis (Fig. 1). Two of them^4,5^ each originally had incomplete data, but when combined, they provided one full data set and were considered as one trial in this analysis. These two studies were conducted by the same authors with the same study design (treatment arm, dose, schedule, and treatment period). Our meta-analysis, therefore, effectively included 5 trials containing 1,947 patients, out of which the data of 1,554 patients were eligible for inclusion in this meta-analyses, 774 patients had been randomized to nab-paclitaxel arm and 780 patients to sb-taxanes arm (sb-paclitaxel 606, docetaxel 174).

### Characteristics of the included studies

The characteristics of the included trials are shown in Table 1. All patients had MBC. From the original studies, the patients’ characteristics (such as age, Eastern Cooperative Oncology Group [ECOG] performance status, estrogen receptor status, progesterone receptor status, etc.) were well balanced between the treatment arms.

**Table 1:** Characteristics of the included studies.

Author (year)	Cancer type	Study Phase	Patients analyzed	Patients per arm	Treatment line	Median age (year)	Drug, dosage (mg/m^2^) and schedule			Study region
Gradishar (2005)^2^	MBC	III	454	229	1^st^ line 42%	53.1	nab-paclitaxel	260	q3w	Russia, Ukraine, USA, Canada, UK
				225	1^st^ line 40%	53.3	sb-paclitaxel	175	q3w	China
Guan (2009)^3^	MBC	II	210	104	1^st^ line 59%	50.0	nab-paclitaxel	260	q3w	
				106	1^st^ line 60%	48.5	sb-paclitaxel	175	q3w	
Gradishar^4,5^ (2009 + 2012)	MBC	II	300	76	1^st^ line 100%	51.7	nab-paclitaxel	300	q3w	Russia, USA
				76		55.4	nab-paclitaxel	100	qw3/4	
				74^†^		53.3	nab-paclitaxel	150	qw3/4	
				74^†^		55.4	docetaxel	100	q3w	
Rugo (2015)* ^6^	MBC	III	783	267^†^	1^st^ line 100%	54.3	nab-paclitaxel	150	qw3/4	USA
				241		54.7	ixabepilone	16	qw3/4	
				275^†^		55.1	sb-paclitaxel	90	qw3/4	
Tamura (2017)^10^	MBC	II	200	100	1^st^ line 73%	60.0	nab-paclitaxel	150	qw3/4	Japan
				100	1^st^ line 70%	58.0	docetaxel	75	q3w	

*Patients in each arm of Rugo (2015) were also administered with bevacizumab 10 mg/kg (q2w) in addition to the study drug.

^†^Selected arms for this study.

Abbreviations: nab, nanoparticle albumin-bound; sb, solvent-based; MBC, metastatic breast cancer; q3w, every 3 weeks; qw3/4, first 3 of 4 weeks; q2w, every 2 weeks.

Two trials^4–6^ were conducted with chemotherapy-naive patients. The other 3 trials^2,3,10^ included both chemotherapy-naive patients and pretreated patients. By reviewing original studies, we found that two trials excluded patients if they received adjuvant chemotherapy with taxanes (sb-paclitaxel or docetaxel) 12 months prior to study enrollment^2,3^, and another trial only included patients who had no history of chemotherapy after confirmation of metastasis^10^. We concluded that these 3 trials would not be any different from the trials with chemotherapy-naive patients.

One trial had three investigational arms and one control arm^4,5^. To avoid double counting of the placebo arm, we chose the nab-paclitaxel arm with 150 mg/m² (qw3/4) (n = 74) as the experimental arm for this analysis. This choice was driven by the fact that the original study indicated the dose was the most effective one compared with the other two nab-paclitaxel dosing regimens of 300 mg/m² (q3w) and 100 mg/m² (qw3/4). Therefore, we thought this nab-paclitaxel dose will more likely be administered in clinical treatments. Another trial had an ixabepilone arm^6^, but we only selected nab-paclitaxel and sb-paclitaxel arms for our meta-analysis.

One trial investigated combined therapy with bevacizumab^6^, while all the other four trials included single taxane-only therapy^2–5,10^. In three trials, the sb-paclitaxel arm was the control group^2,3,6^, and the docetaxel arm was the control group in the other two trials^4,5,10^. Three trials were performed on Western regions^2,4–6^, and the other two trials were conducted on Eastern regions^3,10^.

One trial reported time to progression (TTP)-weeks instead of PFS-months^2^. Considering the fact that TTP and PFS scales are generally similar to one another^3,11^, we used TTP data as the best PFS surrogate for this meta-analysis. TTP-weeks were converted to PFS-months using the multiplier 7/30 (e.g., 23.0 weeks × 7/30 = 5.4 months). Another study presented PFS with 90% CI instead of 95% CI^10^. We converted the 90% CI into 95% CI using methods described by Tierney^12^. Efficacy data, grade ≥ 3 adverse events, and dose discontinuation data for both arms from the five trials are presented in Table 2 and Table 3.

**Table 2:** Grade ≥ 3 adverse events and dose discontinuation rate in overall and subgroup analysis.

		No. of studies	No. of patients	Arm	Patients	Adverse events (%)	Pooled OR (95% CI)	p-value	I^2^ (%)
Neutropenia^2–6,10^	Overall	5	1,541	nab, nab + bev sb, sb + bev	767 774	356 (46.4) 362 (46.8)	0.46 (0.12–1.79)	0.26	96
	Subgroup	4	1,006	nab sb	504 502	222(44.0) 312(62.2)	0.26 (0.09–0.78)	0.02^†^	90
Leukopenia^2,3,6,10^	Overall	4	1,393	nab, nab + bev sb, sb + bev	693 700	143 (20.6) 153 (21.9)	0.77 (0.25–2.44)	0.66	92
	Subgroup	3	858	nab sb	430 428	97(22.6) 132(30.8)	0.51 (0.16–1.59)	0.24	87
Sensory neuropathy^2–6,10^	Overall	5	1,541	nab, nab + bev sb, sb + bev	767 774	139 (18.1) 73 (9.4)	2.44 (1.42–4.20)	0.001^†^	53
	Subgroup	4	1,006	nab sb	504 502	69(13.7) 25(5.0)	2.90 (1.45–5.79)	0.003^†^	48
Fatigue^2,4–6,10^	Overall	4	1,331	nab, nab + bev sb, sb + bev	663 668	61 (9.2) 44 (6.6)	1.34 (0.35–5.22)	0.67	79
	Subgroup	3	796	nab sb	400 396	18(4.5) 17(4.3)	1.25 (0.10–5.16)	0.86	85
Dose discontinuation rate^3–6,10^	Overall	4	1,093	nab, nab + bev sb, sb + bev	541 552	112 (20.7) 85 (15.4)	1.23 (0.68–2.25)	0.49	62
	Subgroup	3	558	nab sb	278 280	42(15.1) 54(19.3)	0.89 (0.55–1.43)	0.64	0

Abbreviations: nab, nanoparticle albumin-bound paclitaxel; sb, solvent-based taxanes; bev, bevacizumab; OR, odds ratio; CI, confidence interval.

^†^Statistically significant when p < 0.05.

Overall includes all studies with adverse events data.

Subgroup includes therapy with nab-paclitaxel vs solvent-based taxanes (sb-paclitaxel and docetaxel).

**Table 3:** Efficacy and Grade ≥ 3 AE between nab-paclitaxel vs sb-paclitaxel or docetaxel each.

Efficacy	Subgroup	No. of studies	OR/HR (95% CI)	p-value	I^2^ (%)
ORR^2–5,10^	nab-paclitaxel vs sb-paclitaxel	2	2.39 (1.69–3.37)	<0.001^†^	0
	nab-paclitaxel vs docetaxel	2	1.38 (0.90–2.11)	0.14	0
DCR^3–5,10^	nab-paclitaxel vs sb-paclitaxel	1	1.89 (1.07–3.35)	0.03^†^	—
	nab-paclitaxel vs docetaxel	2	2.48 (1.39–4.44)	0.002^†^	0
PFS^2–5,10^	nab-paclitaxel vs sb-paclitaxel	2	0.75 (0.62–0.90)	0.002^†^	0
	nab-paclitaxel vs docetaxel	2	0.80 (0.32–1.98)	0.63	88
OS^2–5,10^	nab-paclitaxel vs sb-paclitaxel	2	0.94 (0.78–1.13)	0.53	0
	nab-paclitaxel vs docetaxel	2	0.73 (0.54–0.99)	0.04^†^	0
**Grade ≥ 3 AE**					
Neutropenia^2–5,10^	nab-paclitaxel vs sb-paclitaxel	2	0.70 (0.34–1.42)	0.32	78
	nab-paclitaxel vs docetaxel	2	0.07 (0.03–0.16)	<0.001^†^	0
Leukopenia^2,3,10^	nab-paclitaxel vs sb-paclitaxel	2	0.92 (0.57–1.49)	0.73	0
	nab-paclitaxel vs docetaxel	1	0.15 (0.07–0.33)	<0.001^†^	—
Sensory neuropathy^2–5,10^	nab-paclitaxel vs sb-paclitaxel	2	2.56 (0.62–10.64)	0.20	73
	nab-paclitaxel vs docetaxel	2	3.17 (1.20–8.33)	0.02^†^	51
Fatigue^2,4,5,10^	nab-paclitaxel vs sb-paclitaxel	1	4.82 (1.37–17.02)	0.01^†^	—
	nab-paclitaxel vs docetaxel	2	0.50 (0.04–7.01)	0.60	61
DDR^3–5,10^	nab-paclitaxel vs sb-paclitaxel	1	1.37 (0.30–6.29)	0.68	—
	nab-paclitaxel vs docetaxel	2	0.67 (0.41–1.09)	0.10	0

^†^Statistically significant when p < 0.05. Analyzed using random effects model

Abbreviations: nab, nanoparticle albumin-bound; sb, solvent-based; OR, odds ratio (for ORR, DCR, AEs and DDR); HR, hazard ratio (for PFS and OS); –, not defined.

### Overall analysis

**Figure 2:**
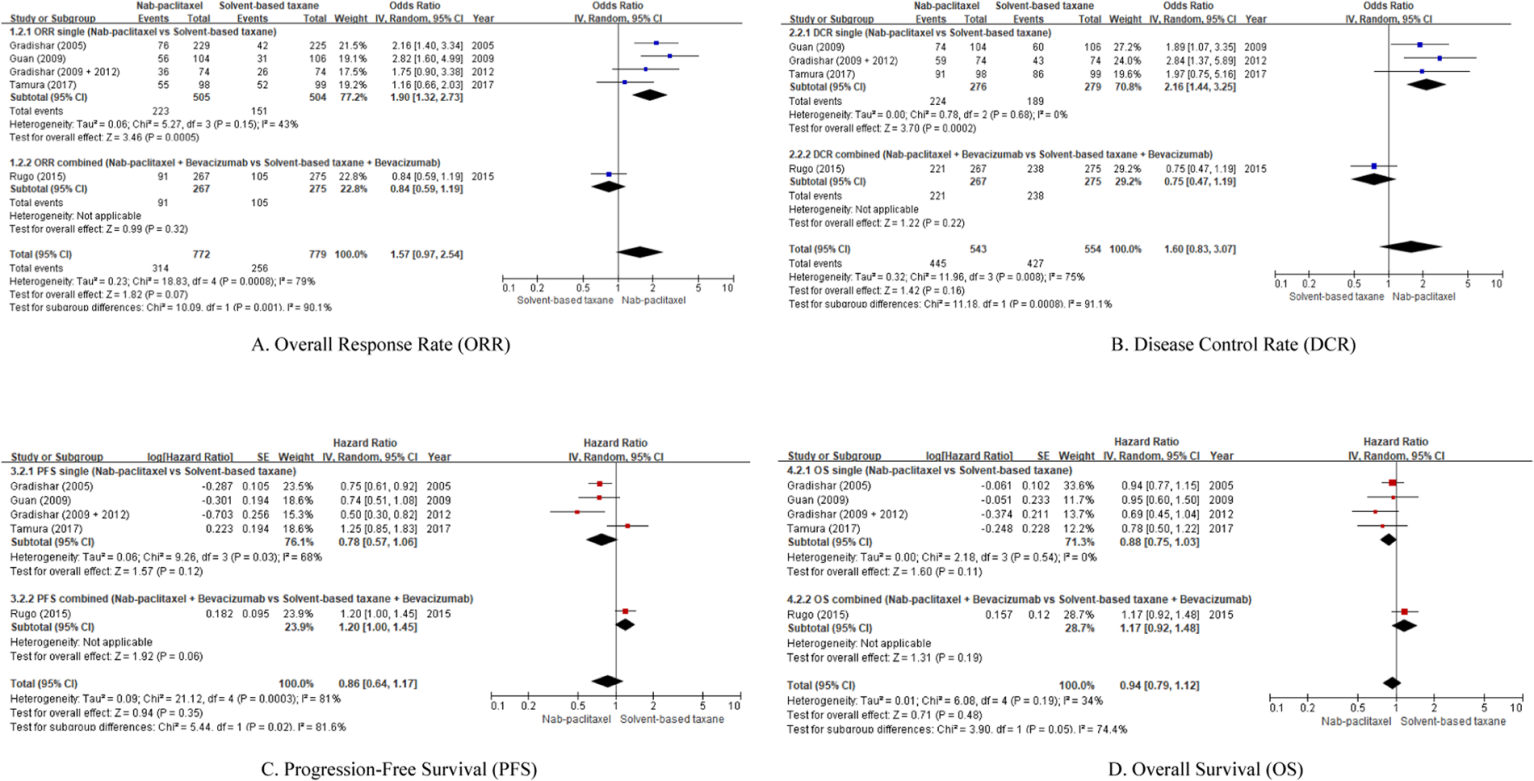
(**A**) Overall Response Rate (ORR). (**B**) Disease Control Rate (DCR). (**C**) Progression-Free Survival (PFS). (**D**) Overall Survival (OS).

All 5 trials were included in the overall meta-analysis. Compared with sb-taxanes, nab-paclitaxel conferred no significant benefit on ORR and DCR (primary outcomes), PFS and OS (secondary outcomes). (p = 0.07, 0.16, 0.35 and 0.48, respectively). These results are shown in the end part of Fig. 2A–D.

In case of grade ≥ 3 adverse events, nab-paclitaxel was inferior to sb-taxanes, since sensory neuropathy was more frequent in nab-paclitaxel arms (OR 2.44, 95% CI 1.42–4.20, p = 0.001), while other AEs, such as neutropenia, leukopenia, fatigue, and DDR were all comparable between the two arms (p = 0.26, 0.66, 0.67, 0.49, respectively).(‘Overall’ rows in Table 2).

### Subgroup analyses

#### Nab-paclitaxel vs sb-taxanes

We conducted subgroup analyses with four single trials (n = 1,012)^2–5,10^ to exclude the confounding effect of bevacizumab, and to secure direct comparison of curative effects between nab-paclitaxel and sb-taxanes. Differently from the overall analysis, ORR was significantly better when nab-paclitaxel was used than when sb-taxanes were used (OR 1.90, 95% CI 1.32–2.73, p = 0.0005, I^2^ = 43%) (Figs. 2A-1.2.1). DCR also showed a similar pattern (3 studies^3–5,10^, n = 558, OR 2.16, 95% CI 1.44–3.25, p = 0.0002, I^2^ = 0%) (Figs. 2B-2.2.1). However, PFS and OS were not significantly better in nab-paclitaxel arm, even though a trend toward a longer PFS and OS were noted in nab-paclitaxel arm (p = 0.12, p = 0.11, respectively) (Figs. 2C-3.2.1 and 2D-4.2.1). The combined therapy with bevacizumab itself showed no superiority of nab-paclitaxel on all 4 efficacy variables (p = 0.32, 0.22, 0.06, 0.19, respectively) (Figs. 2A-1,2,2, 2B-2,2,2, 2C-3,2,2, 2D and 4,2,2).

Grade ≥ 3 neutropenia was less frequent in nab-paclitaxel arm, while sensory neuropathy was more frequent in nab-paclitaxel arm (neutropenia, OR 0.26, 95% CI 0.09–0.78, p = 0.02, I^2^ = 90%; sensory neuropathy, OR 2.90, 95% CI 1.45–5.79, p = 0.003, I^2^ = 48%). Leukopenia, fatigue, and DDR were comparable between the two arms (Table 2).

#### Nab-paclitaxel vs sb-paclitaxel or nab-paclitaxel vs docetaxel

Since there were two types of control arm (sb-paclitaxel arm^2,3^ or docetaxel arm^4,5,10^), we did further subgroup analysis to see whether nab-paclitaxel have superiority to both of the control drugs. When compared to sb-paclitaxel, nab-paclitaxel demonstrated significantly better efficacy in terms of ORR, DCR, and PFS (ORR, OR 2.39, p < 0.001; DCR, OR 1.89, p = 0.03; PFS, HR 0.75, p = 0.002). Nab-paclitaxel also showed superiority to docetaxel on DCR and OS (DCR, OR 2.48, p = 0.002; OS, HR 0.73, p = 0.04). (Table 3)

When compared to sb-paclitaxel, nab-paclitaxel showed comparable toxicities in terms of grade ≥ 3 neutropenia, leukopenia, and sensory neuropathy (p = 0.32, 0.73, 0.20, respectively), but fatigue was significantly more frequent in the nab-paclitaxel arm (OR 4.82, p = 0.01). When compared to docetaxel, neutropenia and leukopenia were significantly less frequent (OR 0.70, p < 0.001; OR 0.15, p < 0.001, respectively), but sensory neuropathy was significantly more frequent (OR 3.17, p = 0.02) in the nab-paclitaxel arm. (Table 3)

#### Nab-paclitaxel vs sb-taxanes by each study region

In the subgroup analyses by study region, nab-paclitaxel showed significantly better results in Western region in terms of ORR and PFS (ORR, OR 2.03, p < 0.001; PFS, HR 0.65, p = 0.03, respectively) compared to sb-taxanes, but not in Eastern region. Nab-paclitaxel showed significantly better results on DCR regardless of the region (Western, OR 2.84, p = 0.005; Eastern, OR 1.91, p = 0.01, respectively) than sb-taxanes. (Table 4)

**Table 4:** Efficacy and Grade ≥ 3 AE between nab-paclitaxel vs sb-taxanes in each study region.

Efficacy	Study region	No. of studies	OR/HR (95% CI)	p-value	I^2^ (%)
ORR^2–5,10^	Western	2	2.03 (1.41–2.92)	<0.001^†^	0
	Eastern	2	1.80 (0.75–4.33)	0.19	79
DCR^3–5,10^	Western	1	2.84 (1.37–5.89)	0.005^†^	—
	Eastern	2	1.91 (1.17–3.12)	0.01^†^	0
PFS^2–5,10^	Western	2	0.65 (0.44–0.96)	0.03^†^	56
	Eastern	2	0.96 (0.58–1.61)	0.88	73
OS^2–5,10^	Western	2	0.85 (0.64–1.13)	0.27	44
	Eastern	2	0.86 (0.62–1.18)	0.35	0
**Grade** ≥ **3 AE**					
Neutropenia^2–5,10^	Western	2	0.38 (0.26–0.54)	<0.001^†^	93
	Eastern	2	0.75 (0.45–1.25)	0.27	91
Leukopenia^2,3,10^	Western	1	0.75 (0.36–1.54)	0.43	—
	Eastern	2	0.41 (0.06–2.79)	0.36	93
Sensory neuropathy^2–5,10^	Western	2	3.13 (1.23–7.94)	0.02^†^	49
	Eastern	2	2.59 (0.60–11.20)	0.20	73
Fatigue^2,4,5,10^	Western	2	0.94 (0.04–23.37)	0.97	92
	Eastern	1	3.03 (0.12–75.28)	0.50	—
DDR^3–5,10^	Western	1	0.65 (0.29–1.48)	0.30	—
	Eastern	2	0.75 (0.43–1.32)	0.32	0

^*^Statistically significant with p < 0.05. Analyzed using random effects model.

Abbreviations: nab, nanoparticle albumin-bound; sb, solvent-based; OR, odds ratio (for ORR, DCR, AEs and DDR): HR, hazard ratio (for PFS and OS); –, not defined.

Nab-paclitaxel showed less frequent neutropenia but more frequent sensory neuropathy (OR 0.38, p < 0.001; OR 3.13, p = 0.02, respectively) than sb-taxanes in the Western region, but not in Eastern region with all grade ≥ 3 AEs.

## Discussion

We conducted a meta-analysis with originally identified 5 RCTs to see whether nab-paclitaxel had a certain benefit over conventional sb-taxanes in terms of efficacy and toxicities in the first-line treatment of MBC. However, the overall analysis results showed no efficacy benefit with worse toxicities of nab-paclitaxel. So, we further conducted subgroup analyses with 4 single taxane RCTs. Through the exclusion of bevacizumab combined therapy, we could find the evidence that nab-paclitaxel was superior to sb-taxanes in terms of ORR and DCR. Concerning grade ≥ 3 AEs, the two study drugs were comparable, since neutropenia was more frequent in the sb-taxane arms, while sensory neuropathy was more frequent in the nab-paclitaxel arms. DDR was also comparable between the two study drugs.

Through the further subgroup analyses within the single taxane subgroup, we could find the evidence that nab-paclitaxel was superior to sb-paclitaxel in terms of ORR, DCR, and PFS. Nab-paclitaxel was also superior to docetaxel on OS. However, the subgroup analyses by study region revealed that these affirmative effects of nab-paclitaxel proved to be justifiable only in the Western region.

The subgroup analyses conducted in our study are meaningful because the US FDA withdrew its accelerated approval of bevacizumab indication as a first-line treatment for MBC in combination with paclitaxel use in December 2010^8^, in the middle of the enrollment period for the Rugo *et al*. study (Oct. 2008-Nov. 2011)^6^, because of the failure to provide evidence of an increase in PFS or OS when nab-paclitaxel or sb-paclitaxel was administered combined with bevacizumab. There were concerns about an overall increase in serious AEs related to bevacizumab as well^8^. Following the withdrawal of FDA approval for bevacizumab indication for MBC patients, although Rugo *et al*. made a protocol amendment to make bevacizumab use optional, 97% of patients still received bevacizumab^6^. From this trial, Rugo *et al*. reported no significant difference in the effect on PFS (primary end point) between the nab-paclitaxel plus bevacizumab arm and the sb-paclitaxel plus bevacizumab arm. They also reported high rates of neurotoxicity (27% of patients had grade ≥ 3 sensory neuropathy), dose reductions (45% by cycle 3), and dose discontinuations (>40% by cycle 5) for the nab-paclitaxel plus bevacizumab arm^6^, suggesting that the combination of bevacizumab with nab-paclitaxel 150 mg/m^2^(qw3/4) was not ideal. Furthermore, the high rate of grade ≥ 3 hematologic adverse events for the nab-paclitaxel plus bevacizumab arm (51%) and the withdrawal of FDA approval of bevacizumab as a first-line treatment for MBC suggests that this regimen will not be pursued in subsequent trials^8,13^.

Even though it might be indirectly inferred from our analyses that nab-paclitaxel monotherapy may have superior efficacy over combined therapy with bevacizumab, this does not necessarily mean that nab-paclitaxel should always be administered by itself. Combination therapy with nab-paclitaxel to increase efficacy is an attractive option for some patients, particularly those with a high tumor burden or those who require rapid disease control. Minckwitz *et al*. noted that various Phase II studies have been conducted evaluating nab-paclitaxel as part of combination therapy with cytotoxics (gemcitabine, capecitabine, carboplatin) and biologics (bevacizumab, trastuzumab) in the treatment of MBC, and although the nab-paclitaxel dosing and schedules varied among these studies, in all cases, the nab-paclitaxel combinations were highly active and well tolerated^14^.

In this analysis, we included DCR as one of the end points. We thought that DCR could be an important criterion influencing the choice of chemotherapy drug for MBC patients. ORR is the sum of complete response (CR) and partial response (PR), while DCR is the sum of additional stable disease (SD) to ORR. So, ORR may be an important index to the patient who hopes “to be getting better,” while DCR may be a critical index to the patient who hopes “not to be getting worse.” Since the 5-year survival of MBC patients is only around 25%^4,13^, the DCR data of a certain drug would be more helpful than the ORR data for a certain group of patients. Moreover, the Southwest Oncology Group reported that DCR might be a better predictor of survival than response rate alone among patients with non-small-cell lung cancer, based on the fact that, for any given treatment, many more patients typically achieve stable disease (SD) or have progressive disease (PD) than achieve a response. They also reported that, although ORR (CR + PR) at week 8 was associated with longer survival (HR 0.61, p < 0.001), DCR (CR + PR + SD) was better for predicting survival (HR 0.45, p < 0.0001)^15^.

For similar reasons, we also analyzed DDR due to unacceptable toxicity or serious AEs as an end point. Considering that 5-year survival is very low among MBC patients, it can be hypothesized that the formal declaration of dose discontinuation by the medical clinic would have a seriously bad impact on patient’s quality of life. DDR due to toxicity would be helpful during the management of MBC patients when choosing between nab-paclitaxel and sb-taxane therapy. In our meta-analysis, DDR was comparable between the two study drugs.

In our regional subgroup analyses, nab-paclitaxel and sb-taxanes were almost comparable in efficacy and toxicities in the Eastern region (only DCR was significantly higher in the nab-paclitaxel arm in the Eastern region). More studies are needed to clarify the reasons of this regional difference, including the genetic characteristics of each regional region.

Our meta-analysis has some limitations. We were not able to take into account the efficacy differences between weekly (qw3/4)^4–6,10^ and q3w^2,3^ of taxane schedule. We also were not able to coordinate the efficacy differences in dosage between docetaxel 100 mg/m^2^ ^4,5^ and 75 mg/m^2^ ^10^. It was because of the small number of eligible RCTs. For the right comparison of nab-paclitaxel with sb-taxane and for the right discrimination of a specific drug’s superiority or inferiority, it should be determined first what dose and what schedules are the best for each study drug, and then we need the actual curative effect data based on their “best” dose and schedules. However, the search of the optimum dose and schedules of nab-paclitaxel and sb-taxanes is still ongoing^13^. Another limitation is that the trial of Rugo (CALGB) did show a PFS and an OS benefit in the TNBC subgroup, however, a subgroup analysis was not performed on the subset of TNBC patients separately. It was because, among 5 RCTs, only 2 studies presented data about the TNBC subset (Rugo 2015 and Tamura 2017). And the results on PFS were just comparable between the study drugs (in Rugo, median PFS 7.4 vs 6.5mon, HR 0.86, 95% CI 0.60–1.25, p = 0.43; in Tamura, median PFS 6.8 vs 7.0mon, HR 1.20, 95% CI 2.90–9.60, p = 0.62).OS data for the TNBC patients was presented only in one study (Tamura 2017), which was also comparable between the study drugs (median OS 27.1 vs 19.3mon, HR 0.56, p = 0.121). Lastly, there existed very high heterogeneities among individual effect sizes, aside from the dose and schedule issue which makes it difficult to interpret the results of this meta-analysis confidently. This “heterogeneity problem” cannot be solved easily, since generally, the number of RCTs are so small in this field, as Higgins *et al*. previously pointed out^16^.

Nevertheless, the high heterogeneity provided an opportunity to probe the consistency of the treatment effects across the single or combined therapy approaches used. Further, all the trials made statements of randomization and presented suitable data for this meta-analysis. Therefore, we deemed methodological quality of these studies not to be a source of heterogeneity.

## Methods

### Literature search

We searched PubMed, Cochrane Library, Ovid MEDLINE, Web of Science, and ClinicalKey, without year and language restrictions. We used (breast neoplasm(s) OR breast cancer) AND (albumin-bound paclitaxel OR nab-paclitaxel OR abraxane OR ABI 007) AND (paclitaxel OR sb-paclitaxel) AND (docetaxel OR taxoids) as a searching algorithm. The last search was updated on May 14, 2018. Reference lists from the relevant full texts were checked to identify other potentially eligible studies. We also manually searched the references of review articles and abstracts from American Society of Clinical Oncology Annual Meetings. Trials were potentially eligible regardless of the study phase, dose schedule, and study region. We followed the Preferred Reporting Items for Systematic Reviews and Meta-Analyses (PRISMA) guidelines with respect to our search strategy and selection process^17^.

### Study selection and data extraction

Two reviewers independently screened the titles and abstracts of all studies identified in the literature search to verify compliance with the inclusion criteria. Disagreements between the two reviewers were resolved by consensus involving a third reviewer. The same reviewers performed data extraction and quality assessment of the included studies.

From each eligible trial, we extracted the following items: study and article characteristics (name of author and journal, year of publication, study phase, treatment line, study region), study region (number of randomly assigned patients in each arm, along with patient age data), study drug (name and dosage, schedule, combination drug if any), efficacy outcomes, adverse event outcomes, and dose discontinuation rate (DDR) due to unacceptable toxicities or adverse events (AEs) for both arms. The PICOS^9^ summary of our study is shown in Table 5.

**Table 5:** PICOS of this study.

P (patients): patients with MBC
I (intervention): nab-paclitaxel
C (comparators): solvent-based taxanes (sb-paclitaxel, docetaxel)
O (outcomes): primary end point; ORR and DCR
secondary end points; PFS, OS, AEs, and DDR
S (study design): RCTs on the efficacy and adverse events comparing nab–paclitaxel-based chemotherapy with solvent-based taxane chemotherapy
MBC: metastatic breast cancer, ORR: overall response rate, DCR: disease control rate, PFS: progression-free survival, OS: overall survival, AE: adverse events, DDR: dose discontinuation rate, RCT: randomized clinical trial

### End points

The primary end points were overall response rate (ORR) and DCR. Secondary end points were PFS, OS, AEs, and DDR.

### Statistical analysis

For binary outcomes (ORR, DCR, AEs, and DDR), odds ratios (ORs) were calculated for each eligible study. For time-to-event outcomes (PFS and OS), hazard ratios (HRs) were calculated for each eligible study to estimate the relative risk among MBC patients receiving nab-paclitaxel vs sb-taxanes. When 95% CI data for PFS or OS were not immediately available from the original studies, we derived them using methods described by Tierney^12^ or NORMSINV function^18^.

We then synthesized the individual OR and HR data across studies using fixed effects (Mantel-Haenszel) or random effects (DerSimonia and Laird) model to obtain pooled effect sizes. Since there were many high heterogeneous variables in our analyses, we mainly adopted random effects model. The presence of statistical heterogeneity was first assessed using Cochran’s Q test (considered significant for p < 0.10)^19^ and quantified using I^2^ statistics^16^. In our study, when high heterogeneities were observed, we conducted subgroup analysis to explore the possible explanations for the observed heterogeneities. After the overall analysis was conducted comparing nab-paclitaxel with bevacizumab vs sb-taxanes with bevacizumab, subgroup analyses were conducted comparing nab-paclitaxel vs sb-taxane without bevacizumab in each group, which compared nab-paclitaxel vs sb-paclitaxel or nab-paclitaxel vs docetaxel. Another subgroup analysis were also conducted to compare nab-paclitaxel vs sb-taxanes based on study regions such as in western region and in eastern region each. All p-values were two-tailed. All the statistical analyses were performed using Revman software V.5.3^20^. We assessed the methodological quality of the eligible trials using Cochrane’s Risk of Bias (RoB) tool^21^. The study protocol was exempted from the requirement for ethical approval by the Ewha Womans University Ethics Committee (IRB No: 163–2).

## Conclusion

Nab-paclitaxel is an effective anti-tumor drug in the first-line treatment of MBC. Using nab-paclitaxel instead of sb-taxanes could significantly improve ORR and DCR with comparable toxicities and DDRs.

## Data Availability

The datasets generated and/or analysed during the current study are not publicly available due to the inclusion of private medical information at our institution, but may be available from the corresponding author on reasonable request.
